# Maternal MicroRNA Profile Changes When LH Is Added to the Ovarian Stimulation Protocol: A Pilot Study

**DOI:** 10.3390/epigenomes7040025

**Published:** 2023-10-06

**Authors:** Fani Konstantinidou, Martina Placidi, Giovanna Di Emidio, Liborio Stuppia, Carla Tatone, Valentina Gatta, Paolo Giovanni Artini

**Affiliations:** 1 Department of Psychological Health and Territorial Sciences, School of Medicine and Health Sciences, “G. d’Annunzio” University of Chieti-Pescara, 66100 Chieti, Italy; fani.konstantinidou@unich.it (F.K.);; 2 Unit of Molecular Genetics, Center for Advanced Studies and Technology (CAST), “G. d’Annunzio” University of Chieti-Pescara, 66100 Chieti, Italy; 3 Department of Life, Health and Environmental Sciences, University of L’Aquila, 67100 L’Aquila, Italy; martina.placidi@univaq.it (M.P.); giovanna.diemidio@univaq.it (G.D.E.); carla.tatone@univaq.it (C.T.); 4 Division of Gynecology and Obstetrics, Department of Clinical and Experimental Medicine, University of Pisa, 56126 Pisa, Italy; paolo.artini@med.unipi.it

**Keywords:** microRNA, oocyte, ovarian stimulation, FSH, LH, Tp53, infertility, IVF

## Abstract

While the use of follicle-stimulating hormone (FSH) in ovarian stimulation for in vitro fertilization (IVF) is an established practice, the use of luteinizing hormone (LH) remains debatable. MicroRNAs (miRNAs) are short, endogenous, non-coding transcripts that control a variety of cellular functions, such as gonadotrophin production and follicular development. The goal of this pilot study was to investigate whether the employment of recombinant LH (rLH) in ovarian stimulation protocols results in changes in the miRNA profiles in human oocytes. Patients were divided into two groups: seven received recombinant FSH (rFSH, 225 IU), and six received rFSH (150 IU) plus rLH (75 IU). MiRNA predesigned panels and real-time PCR technology were used to analyze the oocytes retrieved from the follicular ovarian retrieval. Among the miRNAs evaluated, a series of them evidenced upregulation or downregulation in their expression in the FSH plus LH group compared to the FSH group. Considering the results obtained from the functional and network analysis, the different maternal miRNA profiles in the two groups revealed a differential modulation of pathways involved in numerous biological functions. Overall, based on the pathways associated with most of these maternal miRNAs, the presence of LH may result in a different modulation of pathways regulating survival under the control of a Tp53-related mechanism. Interestingly, among the miRNAs differentially expressed in oocytes of the two groups, we have found miRNAs already investigated at ovarian, follicular, oocyte, and embryonic levels: hsa-miR-484, hsa-miR-222, hsa-miR-520d-5p, hsa-miRNA-17, hsa-miR-548, and hsa-miR-140. Thus, investigation into the role of these miRNAs in oocyte molecular pathways may help determine how LH affects oocyte competence and eventually leads to the clinical improvement of IVF.

## 1. Introduction

MicroRNAs (miRNAs) are single-stranded RNA molecules with nearly 19–25 nucleotides. These RNAs work by regulating and controlling gene expression after transcription or post-transcriptional levels at a mRNA level [1]. Translation of more than 70% of the protein-coding genes is known to be regulated by miRNAs, a role that results in their involvement in both physiological and pathological states [2]. Animal miRNA biogenesis is broken down into various stages. MiRNAs are converted into 22 nt long functional units via the successive cleavage of hairpin structures found in long transcripts by DROSHA/DGCR8 and DICER1. The RNA-induced silencing complex (RISC), which is formed by the association of miRNAs with Argonaute proteins (AGO), mediates gene silencing by binding to partly complementary regions in the 3′ untranslated region of target mRNAs. This causes mRNA deadenylation and/or inhibits translation [3].

The role of miRNAs in the normal development of ovarian follicles and follicle pathogenesis has been extensively investigated and reviewed [4]. During oogenesis, the expression of about 2000 miRNA genes varies in ovarian follicles [5]. It is known that miRNAs present in the follicular fluid and mural granulosa cells of women of different ages varies according to age and also according to the stage of oocyte maturation [6]. Depending on their age and oocyte maturation stage, in vitro fertilization patients have different microRNA profiles in their follicular fluid and mural granulosa cells. In mammalian oocytes and embryos, miRNAs represent one of the three major classes of small non-coding RNAs (sRNAs) including Piwi-interacting RNAs (piRNAs) and endogenous small interfering RNAs (endo-siRNAs) [7].

In mice, while mature oocytes and zygotes have similar expression profiles, there are dynamic changes in miRNA expression during oogenesis [8]. About 60% of the maternally inherited miRNAs are actively degraded during the first cell division and are up-regulated from the two-cell stage onward. Embryo-stage-specific miRNA expression and miRNA-mediated degradation of maternal transcripts during oocyte embryo transition (OET) have been reported in multiple organisms. In humans, a recent study based on RNA-sequencing has shown that OET is associated to activation of miRNA expression in human cleavage stage embryos [9].

A 100-fold-lower miRNA content is found in fully grown oocytes compared to somatic cells, probably as a result of the generally low miRNA accumulation during oogenesis [10]. Since low levels of miRNA were found in rat, hamster, porcine, and bovine oocytes, it can be concluded that mammalian oocytes commonly exhibit miRNA inactivity. Global miRNA levels in humans [9] were comparatively low and generally steady throughout oocyte maturation, indicating that maternal miRNA storage differs from that of mRNA storage, which is very abundant and stored in ribonucleoprotein complexes during oogenesis [11,12,13]. In contrast with mRNA, whose turnover is tailored to oocyte growth, miRNAs seem to be diluted to the point at which they may reduce the ability to control maternal transcriptome [10]. Nevertheless, mRNA components of the miRNA-processing pathway (Drosha, DGCR8, and Dicer) increased during primate oocyte maturation [14,15]. Moreover, Dgcr8-deficient mice oocytes and zygotes either stop growing at the gastrulation stage or develop until the blastocyst stage [16,17], showing the necessity of an active miRNA biogenesis pathway in cellular lineage commitment and cell specification [17,18]. Finally, two extraordinarily abundant maternal miRNAs have been discovered by Kataruka et al. [19] in bovine and porcine oocytes. These miRNAs were found to circumvent the diluting effect and effectively suppress their targets in mammalian oocytes.

Based on these considerations, the role of maternal miRNA would require further investigation. Research regarding their possible modulation under different conditions may be helpful to achieve this goal. In this study, we investigate whether miRNA expression profiles in human oocytes are modulated by the intrafollicular hormonal milieu in ovarian stimulation (OS). The final steps of oogenesis in the in vitro fertilization (IVF) cycle are driven by OS. Follicle-stimulating hormone (FSH) is essential for promoting follicle growth in the ovaries, whereas the function of luteinizing hormone (LH) is still up for debate. During folliculogenesis, LH is necessary for estradiol (E2) generation in the antral stage and further follicular expansion (beyond 10 mm in diameter). During follicle selection, granulosa cells drastically upregulate LHR expression. Thus, the growth and maturity of antral follicles can be divided into those that depend on FSH and those that depend on LH. Antral follicle growth was halted and no preovulatory follicles or corpora lutea were discovered in animals lacking the LH subunit or LHR, proving that LH was necessary for antral follicle maturation and ovulation. The development of LH dependency in antral follicles is a newer idea for follicle selection. The first follicle that expresses LHR in mural granulosa cells and develops LH dependency is thought to have a chance of surviving and maturing into a dominant follicle. LH has been demonstrated to activate the IGF system, promote LHR and aromatase expression, and decrease cell death in mural granulosa cells (tetralogy for follicle selection) in bovine antral follicles. Thus, LH is crucial for follicle selection, antral follicle final maturation, and subsequent ovulation preparation [20].

In OS, a meta-analysis reveals there is no discernible difference between FSH + LH and FSH alone with a GnRH-antagonist protocol [21], although more recent studies [22,23,24] suggest that LH supplementation may be advantageous in some subgroups of women. In a significant retrospective investigation with more than 9000 cycles, it was discovered that LH supplementation increased the cumulative live birth rate in poor ovarian responders more than FSH alone [25].

Furthermore, there is evidence that the molecular environment of the human follicle is altered when LH is added to the ovarian stimulation procedure. According to our earlier research on CCs, differentially expressed genes, evaluated at the periovulation phase, were represented by different gene clusters, each containing transcripts critically involved in growth, development, and communication between cells as well as central processes for the oocyte maturation and its maintenance. Moreover, the presence of LH in OS treatment determines effects on PPARs (peroxisome-proliferator-activated receptors) and their steroidogenic targets in GC [26,27,28].

Recent studies have revealed that supplementation of LH with FSH during OS could modulate gene expression in CCs through miRNA control. Indeed, miRNA expression in CCs is modulated by gonadotropin treatment and correlates strongly with age [29,30]. In addition, the action of LH in modulating the expression of miRNAs in the follicular environment has been documented in animal models [31]. However, to our knowledge, it still remains unknown whether oocyte microRNA storage changes when LH is added to the ovarian stimulation protocol. This would increase knowledge in the possible employment of miRNA as markers of oocyte competence and in the molecular pathways regulated by gonadotropin in the human oocyte.

## 2. Results

### 2.1. Profiling of Patients in the Study Groups

Patient demographics and the clinical profiles of patients are presented in Table 1. The age, basal hormone measurements, and number of metaphase II oocytes collected were not statistically different between FSH and FSH plus LH groups.

### 2.2. Expression Patterns of Oocyte miRNAs in the rFSH-rLH Versus the rFSH Treatment Group

Following q-RT-PCR analysis, a series of microRNAs was detected in the oocytes of patients that had undergone hormonal treatment for IVF purposes with rFSH as well as rFSH-rLH. More specifically, 17 miRNAs were found to be present in the rFSH group and 10 in the rFSH-rLH one (Table 2).

**Figure 1:**
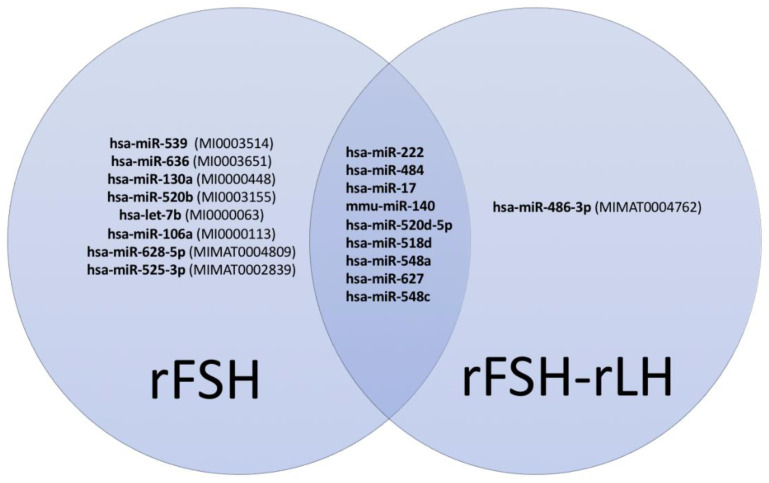
Venn diagram with characterized miRNAs in the studied cohort groups and their individual corresponding miRBase accession IDs.

The oocytes retrieved following both stimulation protocols shared nine of these miRNAs (Figure 1), with three of them—hsa-miR-518d (miRBase, MI0003171), hsa-miR-627 (miRBase, MI0003641), and hsa-miR-548c (miRBase, MI0003630)—being unmodulated in the rFSH-rLH- compared to the rFSH-treated oocytes and six of them being differentially modulated (fold change > 1.4 for up-regulation and <0.7 for down-regulation).

**Figure 2:**
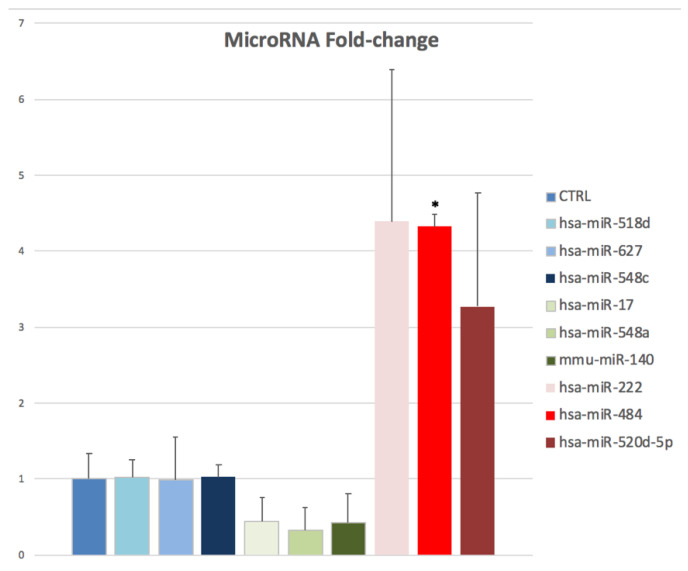
MicroRNA expression (fold-change) histogram of the nine small non-coding RNAs detected in both oocytes of patients treated with both rFSH as well as rFSH-rLH. * indicates a *p*-value < 0.05.

Among the six differentially expressed miRNAs, three showed up-regulation (hsa-miR-484, hsa-miR-222, hsa-miR-520d-5p) and three showed down-regulation patterns (hsa-miR-17, hsa-miR-548a, mmu-miR-140). In particular, hsa-miR-484 evidenced statistically significant up-regulation (mean fold change = 4.33, *p*-value = 0.0011) in the oocytes of patients treated with rFSH-rLH versus those treated with rFSH (Figure 2).

### 2.3. IPA-Inferred Functional and Network Analysis of Oocyte miRNAs Expressed in rFSH and/or rFSH-rLH Treatment Groups

**Figure 3:**
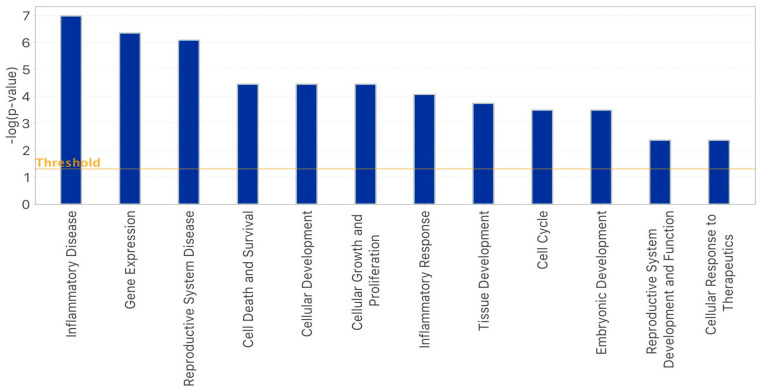
Bar–chart generated via IPA of the principal biological functions regulated by miRNAs expressed in oocytes of both the rFSH and rFSH-rLH groups.

Based on the IPA functional analysis performed as previously described [32], it was highlighted that the nine shared miRNAs between the two OS-derived oocyte groups were involved in numerous process with inflammatory disease, gene expression, and reproductive system disease as the most representative functions (Figure 3).

**Figure 4:**
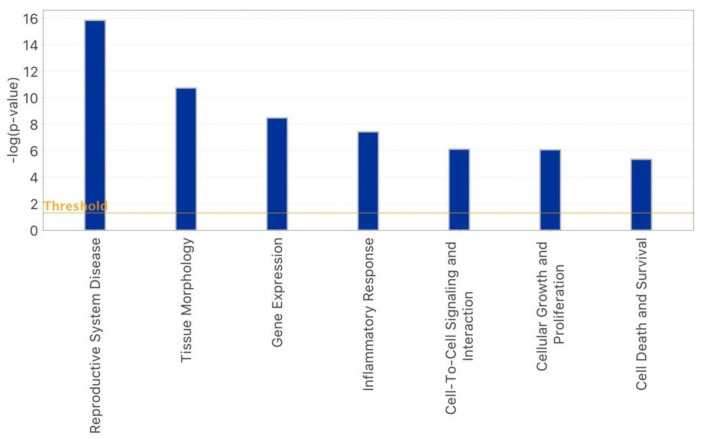
IPA bar–graph with the principal biological functions regulated by oocyte miRNAs detected exclusively in the rFSH group.

**Figure 5:**
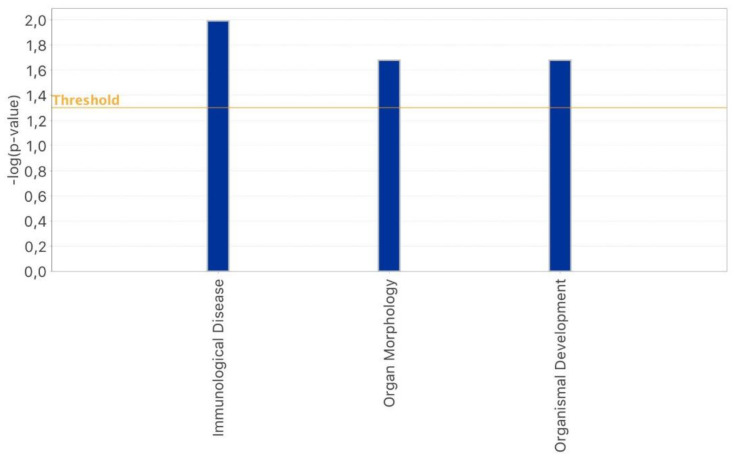
IPA–bar graph with the principal biological functions regulated by oocyte miRNAs detected exclusively in the rFSH-rLH group.

As reported in Figure 1, hsa-miR-539, hsa-miR-636, hsa-miR-130a, hsa-miR-520b, hsa-let-7b, hsa-miR-106a, hsa-miR-628-5p, and hsa-miR-525-3p were only detected in oocytes subjected to OS with rFSH and not in those that were treated with rFSH-rLH. Following a second ingenuity pathway functional analysis, these miRNAs were found to participate only in some of the biological functions reported in Figure 3, including reproductive system diseases, gene expression, inflammatory response, cellular growth and proliferation, and cell death and survival. In addition, the miRNAs detected exclusively in the rFSH group turned out to participate in tissue morphology and cell-to-cell signaling and interaction (Figure 4). On the other hand, hsa-miR-486-3p, specific to the rFSH-rLH group, was predicted to have a key role in organismal development, organ morphology, and immunological disease (Figure 5).

**Figure 6:**
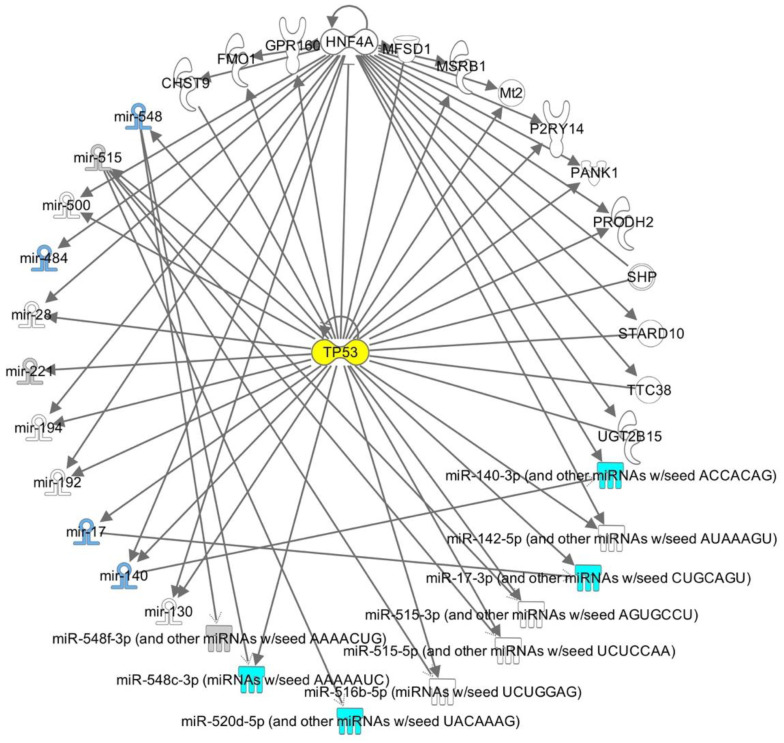
IPA target network analysis of direct relationships between a series of characterized microRNAs in oocytes of both treatment groups and other genes or small non-coding RNAs potentially interacting with them.

IPA was also put into use to better comprehend the networks in which the nine shared miRNAs between the two hormonal treatment groups participate and to unravel potential genes that could target them and regulate their expression. According to the software’s prediction tools and validated reports, the generated top network has as its central node the gene tumor protein p53 (Tp53), considering its vital role in blocking the proliferation of cells with damaged nuclear DNA (Figure 6).

An additional network analysis was performed considering the eight microRNAs—hsa-miR-539, hsa-miR-636, hsa-miR-130a, hsa-miR-520b, hsa-let-7b, hsa-miR-106a, hsa-miR-628-5p, and hsa-miR-525-3p, solely characterized in the rFSH group. The top network generated by IPA resulted involved in the same pathway as the one indicated in Figure 6, sharing the same central node gene, TP53, as well as candidate target genes, except for one, called ORM2. ORM2, known also as orosomucoid 2 gene, encodes a key acute phase plasma protein. This protein has been classified as an acute-phase reactant because following acute inflammation, its levels tend to significantly increase. Although the specific function of this protein has not yet been entirely elucidated, it has been sustained that it could potentially be involved in processes of immunosuppression (Figure S1, Supplementary Materials).

## 3. Discussion

This study evaluated the expression of miRNAs in oocytes from patients undergoing IVF and using solely rFSH or rFSH plus rLH hormonal treatment as their ovarian stimulation strategy. rFSH in combination with a gonadotrophin-releasing hormone (GnRH) analog is one of the several ovarian stimulation regimens used for intracytoplasmic sperm injection (ICSI) or IVF cycles in order to avoid premature LH surges. The topic of whether supplementation with rLH might improve live birth rates arises because GnRH analog deprives the developing follicles of LH. Although there is inadequate evidence to support or refute the use of stimulation regimens for IVF/ICSI cycles that contain both rLH and rFSH [21,33], the addition of LH seems to have positive effects in some subgroups of women [22,23,24]. These controversial clinical results have opened opportunities for molecular studies aimed to investigate potential changes in the human follicle developed under these two different OS regimens.

Our findings demonstrated that oocytes obtained from the two groups show different miRNA expression profiles. Some miRNAs were expressed in both groups, although at different levels, while other miRNAs were found exclusively in the rFSH group. These results could indicate the existence of a possible epigenetic mechanism associated with the expression of small non-coding RNAs behind specific types of gonadotropin administration in an IVF context and corresponding molecular pathways, potentially influencing oocyte competence in female patients. Although miRNAs are a class of non-coding RNA poorly represented in the mature oocyte, the finding that some miRNAs were found exclusively in the rFSH group may reflect changes in epigenetic regulation with potential effects on maternal mRNA storage and activation of embryonic genome [7,8].

Among the six differentially expressed miRNAs, three showed up- (hsa-miR-484, hsa-miR-222, hsa-miR-520d-5p) and three showed down-regulation patterns (hsa-miR-17, hsa-miR-548a, mmu-miR-140) in the oocytes of patients treated with rFSH-rLH versus those treated with rFSH alone. Considering the results obtained from the functional and network analysis, it could be suggested that the differential modulation of these miRNAs between the two OS groups may be related to cell death and survival under the control of a Tp53-related mechanism. The p53 gene family member (TP53 in humans) is a guardian of the female germline and plays a key role in determining the oocyte fate upon damage [34]. P53 is involved in many cellular processes, including steroid hormone regulation, and its role in the pathogenesis of polycystic ovarian syndrome (PCOS)—a complex endocrinologic disorder with increasing risk of infertility, ovulatory disfunction, hyperandrogenism and raised LH:FSH ratio [35]—has been proposed. Recent investigations of increased rates of embryonic implantation failure and miscarriage in women with polymorphisms in the p53 gene could demonstrate the importance of p53 in female fertility and IVF success [36]. It is known that p53 mediates GCs apoptosis and reduces oocyte quality, thus playing an important role in ovarian follicle atresia [37].

In order to discuss the possible effects related to differential expression of miRNAs in the rFSH-rLH group compared to the rFSH one, a literature search took place alongside that of main databases in order to underline the roles associated to these miRNAs in somatic cells and in the reproductive system as well.

Regarding the upregulated miRNAs in the rFSH-rLH group, hsa-miR-520d-5p has been proven to exert an antiapoptotic effect in human dermal fibroblasts exposed to UV irradiation [38]. miR-222 has an oncogenic role in regulating cellular growth of human endometrial carcinoma by suppressing target gene estrogen receptor alpha (ERα) and thus, its overexpression promotes cell proliferation and inhibits apoptosis [39].

Regarding miRNAs downregulated in the rFSH-rLH group, it has been demonstrated that the silencing of hsa-miR-17 regulates angiogenesis in multiple settings [40], can suppress cell proliferation, and promotes cell apoptosis in laryngeal squamous cell carcinoma. On the other hand, it has been seen that overexpression of miR-548a-5p can hamper cell apoptosis [41], suggesting a pro-apoptotic effect in our specific sample groups considering its detected down-expression. Lastly, it has been reported that overexpression of miR-140, found downregulated in our cohort of samples, reduces proliferation, migration, and invasion and promotes apoptosis of colorectal cancer cells [42]. The different roles of such miRNAs in apoptosis could support the idea that the presence of LH in OS results in a different modulation of pathways regulating survival that deserves better investigation.

Concerning the ovarian functions of major involvement, we found that hsa-miR-484, demonstrating a statistically significant up-regulation (mean fold change = 4.33, *p*-value = 0.0011) in our analysis, is known to repress the proliferation of granulosa cells (GCs) and promote apoptosis in GCs due to oxidative-stress-induced ovarian dysfunction [43,44]. Moreover, this miRNA was disclosed to directly target YAP1 mRNA, inducing mitochondrial dysfunction and promoting the apoptosis of GCs, as a possible mechanism underlying diminished ovarian reserve [43]. Interestingly, according to oxidative stress models of mouse ovaries and GCs, it was further reported that oxidative stress could potentially cause some epigenetic alterations that ultimately resulted in modifications in the expression levels of miR-484. Overexpressing miR-484 was found, in fact, to repair mitochondrial activities and raise apoptosis under oxidative stress in GCs. Additionally, miR-484 knockdown contributed to an increase in GC growth and a decrease in intercellular ROS levels. The putative mechanisms might be explained by miR-484’s potential control over mitochondrial processes and the mitochondria-related apoptotic pathway.

In miRNA studies, miR-222 has been observed in different stages of follicle development [44]. In a recent investigation on rats and humans, miR-222 was found to be related to the etiology of PCOS [45]. The modulation of androgen (ARs) and ERs during follicle growth and in hormone-responsive malignancies is known to be significantly influenced by miRNAs [46]. Rat GCs are the primary source of ARs mRNA expression, and as follicle development progresses, so does the expression of these receptors. According to recent research, androgens suppress the expression of miR-222 in ovarian follicles by targeting p27/kip1 in hyperandrogenic DHT rats that have down-regulated granulosa cell AR. In the same PCOS model, miR-222 was discovered to be expressed in the GCs of early-stage follicles as well as in theca cells, where it likely regulates the ERs. Its expression pattern alters with greater follicular growth and antrum formation. Then, miR-222 expression is exclusively seen in theca in the cystic follicle, indicating that miR-222 controls AR expression and, consequently, paracrine regulation [46]. Human studies revealed that the expression of miR-222 was significantly upregulated in PCOS tissues and miR-222-inhibitor-induced apoptosis and cell cycle arrest in GCs [47]. Interestingly, p27 Kip1 was found to be a target gene of miR-222 [47]. These findings support the hypothesis that miR-222 is involved in the progression of PCOS. Besides the ER-alpha regulation by miR-222, some in vitro and in vivo studies in cows have reported an inverse correlation between LHCGR and miR-222 expression in granulosa cells [31]. On the basis of these studies, it could be stated that the upregulation of miR-222 in oocytes of FSH-LH group deserves further investigations to understand its role in oocyte competence.

Moreover, Battaglia et al. reported that miR-520d-5p is specifically expressed in oocytes and absent in follicular fluid. MiR-520d-5p has been also reported to play a role in early embryo development during the maternal RNAs degradation associated with the maternal–zygotic transition [48,49,50]. Thus, the upregulation of this miRNA, as detected in the oocytes of the rLH group, may account for a differential regulation of the maternal mRNA degradation.

The miR-17-92 miRNA cluster is present at considerably high levels in oocytes and embryos, with a transiently lower expression at the two-cell stage. It has also been reported that the ablation of this miRNA cluster in female mice germ cells results in disrupted oogenesis, increased oocyte degradation, and follicular atresia as well as in elevated expression of genes involved in follicular atresia and the mitochondrial apoptotic pathway and, therefore, promotes apoptosis. By specifically targeting Bmf in mouse ovaries with conditional knockout for the miR-17-92 cluster, it was demonstrated that miR-19a regulated oogenesis at the post-transcriptional stage [51]. Additionally, miR-17-92 gene clusters suppress PTEN expression, which is noticeably changed in PCOS. Therefore, the observed downregulation of hsa-miR-17, alongside the anti-apoptotic effect exerted by some of the other modulated miRNAs, following treatments with rFSH-rLH compared to administration of solely rFSH in our cohort of samples, could suggest that further attention should be focused on the presence of LH in ovarian stimulation protocols, which could result in a different modulation of pathways regulating survival that deserves a better investigation.

MicroRNA hsa-miR-548a, which was found to be down-expressed in the rFSH-rLH group, belongs to a primate-specific miRNA gene family with 69 members. According to Son et al. [52], miRNA-548 may play a role in the etiology of preterm birth by regulating high-mobility group box 1 and other processes. miR-548a-3p was found to be expressed in human oocytes and not in other follicle compartments [49]. According to Battaglia et al. (2017), miR-548 genes may be crucial for the maintenance of oocyte quality and deserve investigation in future studies [50].

Among the miRNA we found downregulated in oocytes of the rFSH-rLH group, miR-140 is known to be involved in the repression of Smad3 in the murine multipotential mesenchymal cells blocking the TGF pathway [53]. This pathway is crucial for the regulation of trophoblast differentiation in peri-implantation mouse embryos [54,55,56].

Finally, the microRNA miR-486-3p, characterized exclusively in the rLH-combined regimen, has been reported to have central roles in several types of oncological and non-oncological conditions, such as breast, liver, and lung cancers as well as the autism spectrum and metabolic syndromes. The miR-486 cluster is considered one of the top clusters acting as potential tumor suppressors, with miR-486-3p resulting involved in cell proliferation, differentiation, and apoptosis and being defined as an important functional regulator with aberrant expression in several pathological conditions [57]. In cervical cancer patients, it has been found to repress cell proliferation and metastasis [58]. More specifically, though, it has been further demonstrated that the expression of mouse miR-486-3p (mmu-miR-486-3p), identical to hsa-miR-486-3p, was reported to be significantly increased during the peri-implantation period, with levels significantly higher at implantation than at non-implantation sites, suggesting an active involvement in embryo implantation [59].

## 4. Materials and Methods

### 4.1. Patient Population

The study included 24 MII oocytes (24 GonalF and 20 RFSH-rLH) originating from 13 women with a median age of 34.8 ± 1.74 years randomly chosen from among those with supernumerary oocytes at the Center of Infertility and Assisted Reproduction of the Department of Clinical and Experimental Medicine of Pisa from June 2019 to December 2020. The study was conducted according to the guidelines of the Declaration of Helsinki and approved by the Ethics Committee (CTO, Clinical Trial Office) of Azienda Ospedaliero Universitaria Pisana (AOUP) (protocol code 35,105, approved on 13 June 2019).

The inclusion criteria for the enrolment of patients were as follows: age younger than 38 years; hormonal measurements considered normal (FSH ≤ 14 IU/mL, LH ≤ 12 UI/mL, thyroid stimulating hormone ≤ 3.0 mcIU/mL and prolactin < 31 mg/mL); no hormonal contraceptive in the last 3 months; and tubal factor infertility, unexplained infertility, or male factor infertility. Women who presented with unilateral or bilateral hydrosalpinx, severe (stages III or IV) endometriosis, polycystic ovary syndrome, ovarian failure, or any ovarian or intrauterine anatomical modification were disqualified.

### 4.2. Ovarian Stimulation (OS)

Clinical guidelines from the institution were used to oversee and manage the IVF treatments. The participants in this study were prospectively enrolled to receive any of those two ovarian stimulation procedures. Patients were randomly assigned to either the recombinant FSH regimen or the recombinant FSH plus recombinant LH protocol in a 1:1 ratio. To achieve OS, a gonadotropin-releasing hormone (GnRH) antagonist protocol was used to administer FSH (Gonal-F, recombinant FSH, 225 IU, Merck Serono, Darmstadt, Germany) to 7 patients and FSH plus LH (Pergoveris, recombinant FSH, 150 IU + recombinant LH, 75, IU, Merck Serono, Darmstadt, Germany) to 6 patients. When the lead follicle measured 12–14 mm, patients were administered 0.125 mg/d of Cetrorelix (Cetrotide, Merck, Darmstadt, Germany), a GnRH antagonist, to avoid an early surge in LH. The maturity of the last oocyte was achieved by giving a dosage of 250 mg of recombinant hCG (Ovitrelle, Merck, Darmstadt, Germany) when the diameter of 1–2 follicles measured 17 mm. Patients had a transvaginal follicular aspiration to collect the oocytes after 36 h.

### 4.3. Oocyte Collection

Cumulus enclosed oocytes retrieved during pickup were isolated from the follicular fluid and incubated at 37 °C and 6% CO_2_ for 2 h in HEPES-buffered medium (Cook, Brisbane, Australia) The cumulus oophorus mass was mechanically dissociated and exposed to enzymatic action of 80 IU/mL hyaluronidase (BioCare Europe S.r.l.—Irvine Scientific, Milan, Italy). After denudation, oocytes were examined to assess maturational stage. The samples are washed with PBS and stored at −80 °C until use for experimental analysis. Only cumulus cells collected from mature metaphase II stage (MII) oocytes were included in the study.

### 4.4. RNA Extraction from Oocytes

Collected pools of oocytes were conserved in lysis buffer at −80 °C until the moment of nucleic acid extraction. Total RNA, including miRNAs, was manually isolated using the Nucleospin miRNA kit (Macherey-Nagel, Milan, Italy) according to the manufacturer’s instructions. Quantity and quality of total RNA were assessed by Qubit 2.0 (Invitrogen, Monza, Italy).

### 4.5. Characterisation and Expression Profiles of microRNAs in Oocytes of Women Undergoing IVF

**Figure 7:**
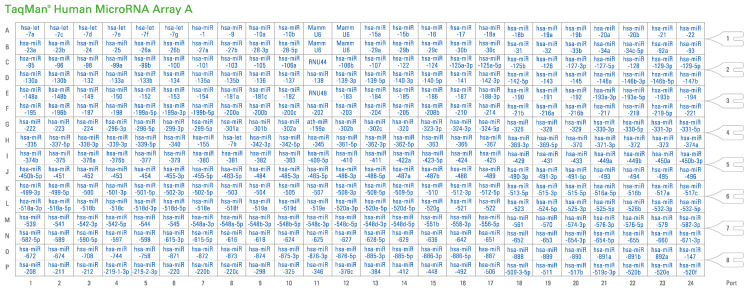
Overview of the TaqMan Human MicroRNA Array A assays (Applied Biosystems, Foster City, CA, USA).

MicroRNA analysis in oocytes retrieved from women undergoing IVF was carried out by TaqMan™ Array Human MicroRNA A Cards v2.0 (Applied Biosystems, Foster City, CA, USA) (Figure 7).

The TaqMan Array Human MicroRNA A Card v2.0 contains 384 TaqMan MicroRNA Assays enabling accurate quantitation of 377 human microRNAs through real-time PCR technology. Three TaqMan MicroRNA Assay endogenous controls are included to aid in data normalization, and one TaqMan MicroRNA Assay not related to humans is included as a negative control. Following an initial Megaplex RT Reaction with Megaplex RT primer Pool A and TaqMan MicroRNA Reverse Transcription kit in order to reverse-transcribe miRNAs (Applied Biosystems, Waltham, MA, USA), a preamplification reaction of the obtained complementary DNAs (cDNAs) also took place through use of the TaqMan PreAmp Master Mix 2X and Megaplex PreAmp Primers pool A (Applied Biosystems, Waltham, MA, USA). At that point, the preamplified products were diluted in 0.1× TE at pH 8.0 and mixed with the TaqMan™ Universal Master Mix II, no UNG (Applied Biosystems, Waltham, MA, USA) and nuclease-free water. They were then loaded into the TaqMan™ Array Human MicroRNA A plates and processed via qRT-PCR using an ABI 7900HT sequencing detection system (Life Technologies, Carlsbad, CA, USA). All experiments were performed in triplicate. MiRNAs were initially characterized for each treatment group, and subsequently, their expression profile between the two groups was estimated. A gene was evaluated as differentially expressed in oocytes of women treated with rFSH-rLH compared to those treated with rFSH when showing a fold change >1.4 or <0.7 (DataAssist Software, Thermo Fisher Scientific, Waltham, MA, USA). Statistical significance of the miRNAs’ expression in these conditions was assessed via *t*-test, accepting *p*-values inferior to 0.05.

### 4.6. Ingenuity Pathway Analysis

Characterised miRNAs were analyzed by Ingenuity Pathway Analysis (IPA) software (Ingenuity Systems, Redwood City, CA, USA) in order to evidence the biological functions of major interest and potential molecular networks in which they are actively involved. IPA functional and network analyses were conducted as previously reported [32].

A flowchart indicating patient recruitment and the experimental process in its totality is provided in Supplementary Materials (Figure S2).

## 5. Conclusions

In clinical practice, LH is added to ovarian stimulation in an effort to increase the oocyte competence [60]. Although FSH plays an important role in the regulation of follicle recruitment and growth, LH is mandatory for further follicular growth and follicular production in the antral stage. In contrast to FSH receptor expression, LH receptor expression is significantly induced in GCs during follicle selection [20]. The present study provides evidence that LH-related molecular mechanisms include changes in the miRNA profile of mature oocytes, and it allows us to conclude that the action of LH in modulating the expression of miRNAs in the follicular environment can be revealed not only at GCs and follicular fluid but also at the oocyte level. This supports the importance of LH for oocyte maturation at the conclusion of the follicular phase [61] and is consistent with the observation that LH supplementation influences the intrafollicular steroid milieu, mimicking physiological conditions [62].

Based on bioinformatics analysis and literature review, differences in the maternal miRNA expression profiles between the rFSH and rFSH-rLH groups revealed differential modulation of pathways involved in numerous biological functions. Based on the pathways associated with most of these maternal miRNAs, the presence of LH in OS may result in a different modulation of pathways regulating survival. Interestingly, among the miRNA differentially expressed in oocytes of the two groups, we have found miRNAs already investigated in the ovary, follicles, oocytes and embryos. These include hsa-miR-484, hsa-miR-222, hsa-miR-520d-5p, hsa-miRNA-17, hsa-miR-548, and hsa-miR-140. Thus, the investigation of the role of these miRNAs in oocyte molecular pathways may help determine how LH affects oocyte competence and eventually leads to clinical improvement.

The present study’s limited patient population is a drawback; as a result, it must be regarded as a pilot study. However, this is merely preliminary screening research in which an extensive variety of miRNAs was examined. In future investigations, it would be interesting to examine the genes controlled by the differentially expressed miRNAs in order to validate the results reported. Thus, by thoroughly elucidating the underlying mechanisms, it could become feasible to unravel the specific manner in which our current findings may possibly result in clinical improvement in an IVF setting.

## Figures and Tables

**Table 1:** Patient demographics and baseline characteristics.

	r-hFSH	r-hFSH + r-hLH	*p* Value
No. of patients	7	6	NS
Age (y)	33.7 ± 3.1	35.3 ± 2.1	NS
FSH (IU/mL)	7.7 ± 2.1	8.1 ± 1.8	NS
LH (IU/mL)	5.7 ± 2.3	5.1 ± 2.1	NS
AMH (ng/mL)	1.9 ± 0.9	1.7 ± 0.7	NS
AFC	13.7 ± 2.5	12.9 ± 2.3	NS
No. supernumerary oocytes MII	24	20	NS

**Table 2:** MicroRNAs characterized in oocytes of rFSH and rFSH-rLH treatment protocols.

No. of microRNAs	r-hFSH	r-hFSH + r-hLH
1	hsa-miR-548c	hsa-miR-548c
2	hsa-miR-222	hsa-miR-222
3	hsa-miR-484	hsa-miR-484
4	hsa-miR-539	hsa-miR-486-3p
5	hsa-miR-636	hsa-miR-17
6	hsa-miR-17	mmu-miR-140
7	hsa-miR-130a	hsa-miR-520d-5p
8	mmu-miR-140	hsa-miR-518d
9	hsa-miR-520b	hsa-miR-548a
10	hsa-let-7b	hsa-miR-627
11	hsa-miR-106a	-
12	hsa-miR-520d-5p	-
13	hsa-miR-518d	-
14	hsa-miR-548a	-
15	hsa-miR-628-5p	-
16	hsa-miR-525-3p	-
17	hsa-miR-627	-

## Data Availability

All original images and data are contained within the article.

## Supplementary Materials

The following supporting information can be downloaded at: https://www.mdpi.com/article/10.3390/epigenomes7040025/s1, Figure S1: IPA target network analysis of direct relationships between the eight microRNAs, solely characterized in the oocytes of the rFSH group, and other genes or small non-coding RNAs detected in the same pathway; Figure S2: Flowchart describing the patient recruitment, sample collection and overall experimental process followed.
