# Supplementary material for: Maternal MicroRNA Profile Changes When LH Is Added to the Ovarian Stimulation Protocol: A Pilot Study
Source: Epigenomes. 2023 Oct 6;7(4):25. doi: 10.3390/epigenomes7040025 (PMC10594432; doi:10.3390/epigenomes7040025)
Supplement: Supplementary file 1 [file epigenomes-07-00025-s001.zip › epigenomes-2556580-supplementary.pdf]

Supplementary Materials

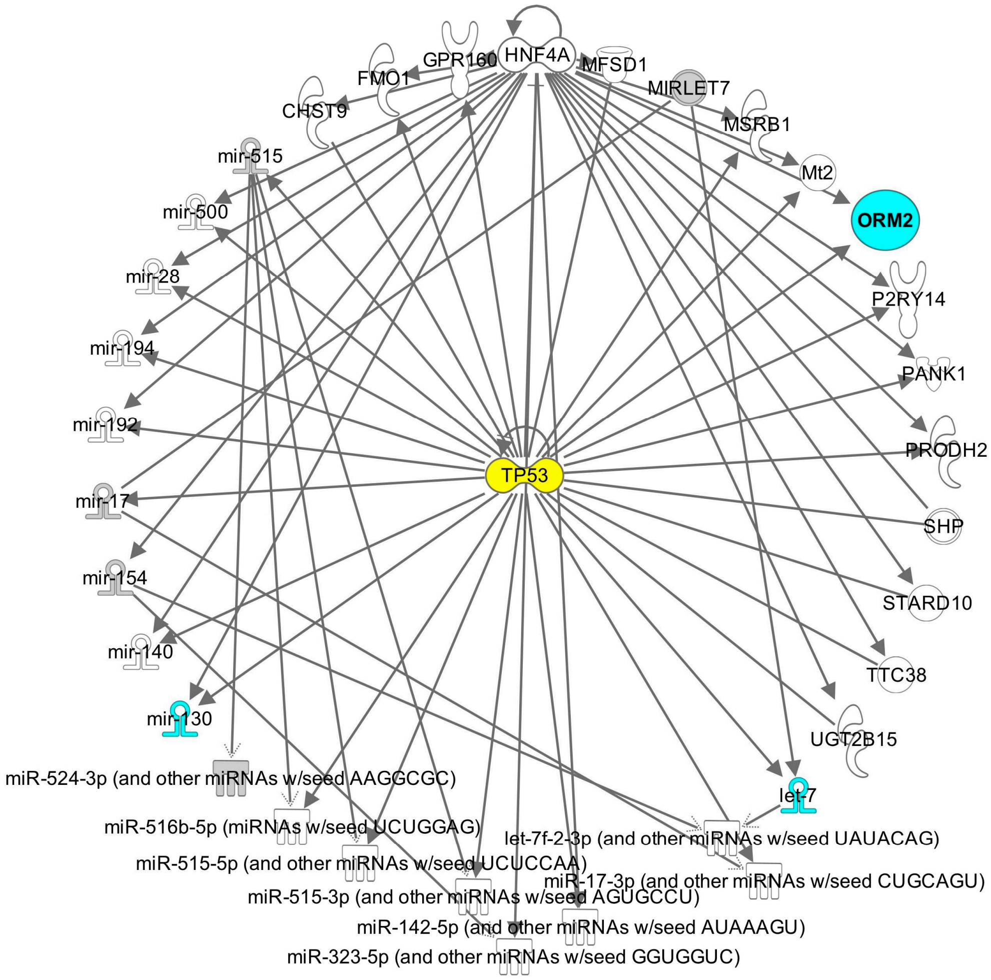

Figure S1. IPA target network analysis of direct relationships between the eight microRNAs, solely characterized in the oocytes of the rFSH group, and other genes or small non-coding RNAs detected in the same pathway.

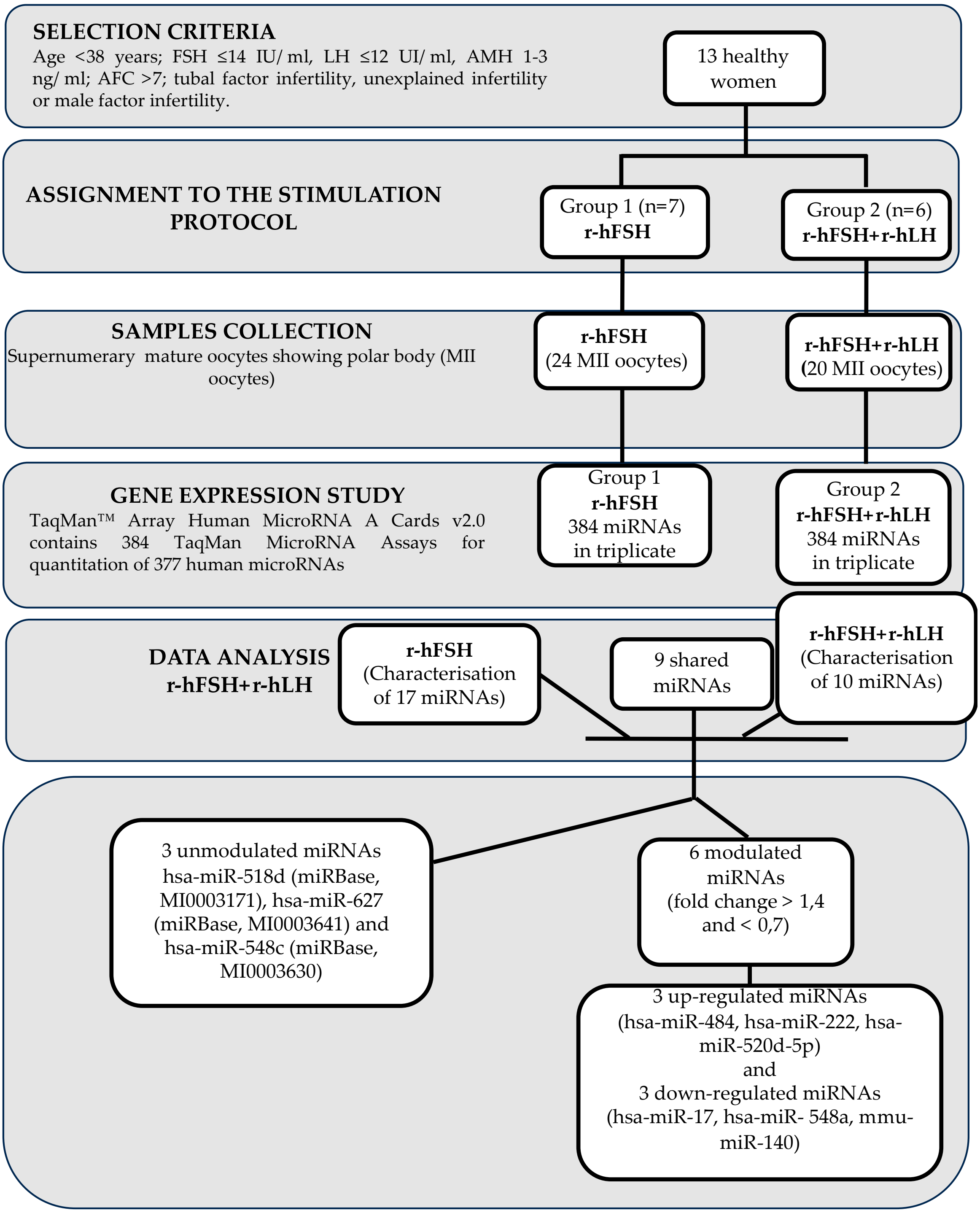
SELECTION CRITERIA
Age <38 years; FSH ≤14 IU/ml, LH ≤12 UI/ml, AMH 1-3 ng/ml; AFC >7; tubal factor infertility, unexplained infertility or male factor infertility.
13 healthy women
ASSIGNMENT TO THE STIMULATION PROTOCOL
Group 1 (n=7) r-hFSH
Group 2 (n=6) r-hFSH+r-hLH
SAMPLES COLLECTION
Supernumerary mature oocytes showing polar body (MII oocytes)
r-hFSH (24 MII oocytes)
r-hFSH+r-hLH (20 MII oocytes)
GENE EXPRESSION STUDY
TaqMan™ Array Human MicroRNA A Cards v2.0 contains 384 TaqMan MicroRNA Assays for quantitation of 377 human microRNAs
Group 1 r-hFSH 384 miRNAs in triplicate
Group 2 r-hFSH+r-hLH 384 miRNAs in triplicate
DATA ANALYSIS r-hFSH+r-hLH
r-hFSH (Characterisation of 17 miRNAs)
9 shared miRNAs
r-hFSH+r-hLH (Characterisation of 10 miRNAs)
3 unmodulated miRNAs hsa-miR-518d (miRBase, MI0003171), hsa-miR-627 (miRBase, MI0003641) and hsa-miR-548c (miRBase, MI0003630)
6 modulated miRNAs (fold change > 1,4 and < 0,7)
3 up-regulated miRNAs (hsa-miR-484, hsa-miR-222, hsa-miR-520d-5p) and 3 down-regulated miRNAs (hsa-miR-17, hsa-miR- 548a, mmu-miR-140)

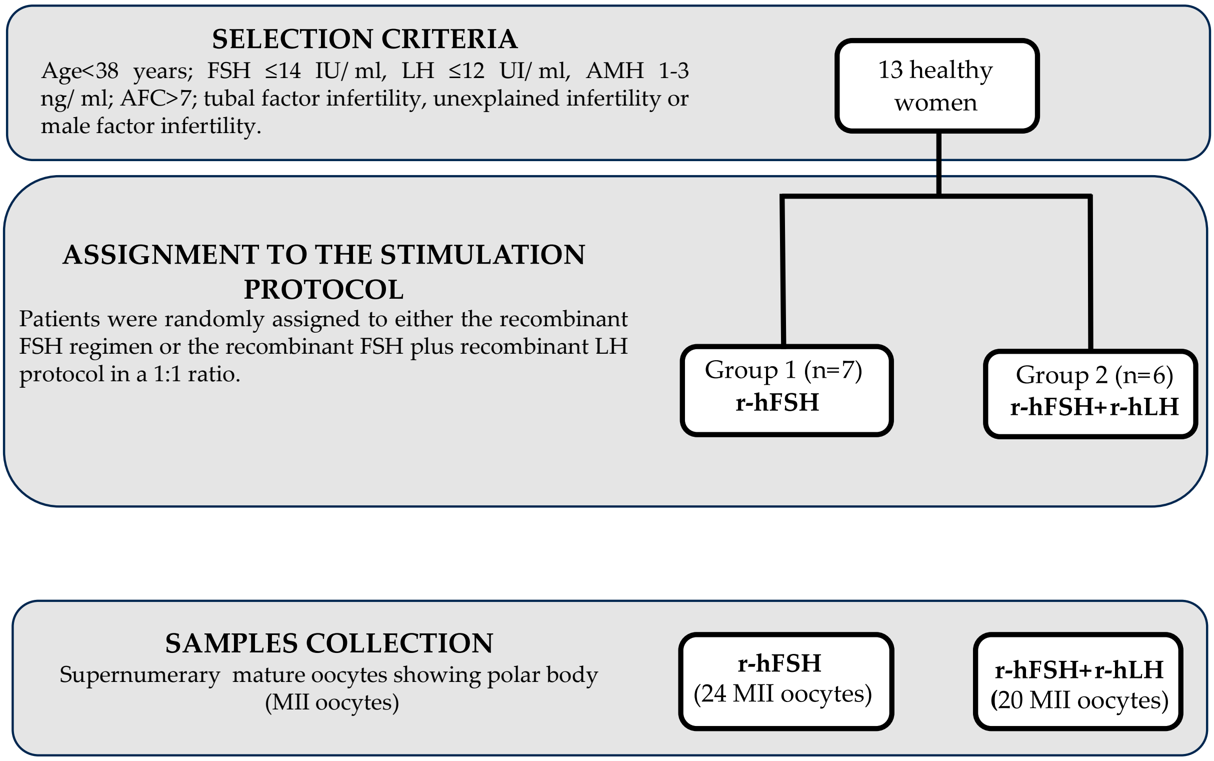

Figure S2. Flowchart describing the patient recruitment, sample collection and overall experimental process followed.
